# Genome Wide Association Study of Age at Menarche in the Japanese Population

**DOI:** 10.1371/journal.pone.0063821

**Published:** 2013-05-07

**Authors:** Chizu Tanikawa, Yukinori Okada, Atsushi Takahashi, Katsutoshi Oda, Naoyuki Kamatani, Michiaki Kubo, Yusuke Nakamura, Koichi Matsuda

**Affiliations:** 1 Laboratory of Molecular Medicine, Human Genome Center, Institute of Medical Science, The University of Tokyo, Tokyo, Japan; 2 Center for Genomic Medicine, The Institute of Physical and Chemical Research (RIKEN), Kanagawa, Japan; 3 Division of Rheumatology, Immunology, and Allergy, Brigham and Women's Hospital, Harvard Medical School, Boston, Massachusetts, United States of America; 4 Program in Medical and Population Genetics, Broad Institute, Cambridge, Massachusetts, United States of America; 5 Department of Obstetrics and Gynecology, Faculty of Medicine, The University of Tokyo, Tokyo, Japan; 6 Departments of Medicine and Surgery, and Center for Personalized Therapeutics, The University of Chicago, Chicago, Illinois, United States for America; The University of Tokyo, Japan

## Abstract

Age at menarche (AAM) is a complex trait involving both genetic and environmental factors. To identify the genetic factors associated with AAM, we conducted a large-scale meta-analysis of genome-wide association studies using more than 15,000 Japanese female samples. Here, we identified an association between SNP (single nucleotide polymorphism) rs364663 at the *LIN28B* locus and AAM, with a P-value of 5.49×10^−7^ and an effect size of 0.089 (year). We also evaluated 33 SNPs that were previously reported to be associated with AAM in women of European ancestry. Among them, two SNPs rs4452860 and rs7028916 in *TMEM38B* indicated significant association with AAM in the same directions as reported in previous studies (P = 0.0013 with an effect size of 0.051) even after Bonferroni correction for the 33 SNPs. In addition, six loci in or near *CCDC85A*, *LOC100421670*, *CA10*, *ZNF483*, *ARNTL*, and *RXRG* exhibited suggestive association with AAM (P<0.05). Our findings elucidated the impact of genetic variations on AAM in the Japanese population.

## Introduction

Age at menarche (AAM), the onset of the first menstrual period in girls, is considered as a landmark of female pubertal development. Menarche generally occurs after a series of complex neuroendocrine events leading to full activation of the hypothalamic-pituitary-gonadal axis [1]. Menarche is associated with physical, emotional, and social development [2]. In addition, AAM was shown to be associated with the risk of various diseases. Early AAM is reported to be one of the significant risk factors for depression [3], eating disorders [4], obesity [5], diabetes [6], breast cancer [7], and coronary heart disease [8]. On the other hand, late AAM has been associated with osteoporosis [9] and taller adult stature [10]. Therefore, the identification of loci contributing to variation in AAM could lead to a better understanding of a wide range of phenotypes.

AAM is known to be a complex trait determined by an array of genetic and environmental variables [11]–[13]. Twin and family studies suggest a significant genetic contribution to AAM with a heritability of more than 50% [12], [14]. Several genetic variations within candidate genes such as the estrogen receptor genes (*ESR1* and *ESR2*) [15], [16], *CYP19A1* [17], and the *SHBG* gene [18] were shown to be associated with AAM. To date, a number of genome-wide linkage analyses [14], [19], [20] and genome-wide association studies (GWAS)[21]–[25] for genes underlying variation in AAM have been performed. In 2009, the association of genetic variations in *LIN28B* with AAM was identified by four independent groups. Currently, more than 30 loci have been shown to be significantly associated with AAM. However, most of these studies were conducted using women of European ancestry. Here we performed a large scale meta-analysis of GWAS using more than 15,000 Japanese female samples.

## Results

A total of 15,495 Japanese female subjects from four GWAS using different SNP genotyping systems were enrolled in this analysis. Characteristics of samples and genotyping methods are summarized in **Table 1**. All the subjects were of Japanese origin and obtained from the Biobank Japan Project [26]. Samples consist of patients that were classified into 33 disease groups. The average and S.D. of AAM in each disease cohort is shown in **Table S1**. Some diseases such as breast cancer and osteoporosis are likely to be associated with early or late AAM, as reported previously [7], [9]. We also found that AAM was negatively associated with birth year (p<0.0001). Thus we used disease status and birth year as covariates in this study. Genotyping was performed with over 500,000 SNP markers using Illumina HumanHap 550 Genotyping BeadChip, Illumina610-Quad Genotyping BeadChip, or Illumina Omni Express (Illumina, CA, USA). We applied stringent quality control criteria as mentioned in the methods section. We also conducted principal component analysis [27] to evaluate potential population stratification. To extend the genomic coverage and conduct meta-analysis, we subsequently performed a whole-genome imputation of the SNPs, using HapMap Phase II genotype data [28]. After the imputation, we performed SNP quality control (minor allele frequency≥0.01 and an imputation score (*Rsq* value by MACH software [29])≥0.7) and found that more than two million autosomal SNPs satisfied these criteria.

**Table 1:** Characteristics of study population.

Cohorts	Number of Samples	Source	Platform	Inflation factor	SNP number	Age (S.D.)	Diseases
Cohort1	11,454	BioBank Japa n	Illumina HumanHap 610	1.052	2,263,308	59.81+−13.25	Cancer(Colorectal, breast, lung, gastric, pancreas, liver, cholangiocarcinoma), diabetes mellitus, myocardial infarction, brain infarction, arteriosclerosis obliterans, Arrythimia,drug eruption, liver cirrhosis, amyotrophic lateral sclerosis, osteoporosis, fibroid, Rheumatoid arthritis, and drug response
Cohort2	941	BioBank Japan	Illumina HumanHap 550	1.001	2,220,799	47.01+−15.04	Cancer (cervival, uterus, esophageal, hematopoietic, cholangiocarcinoma, ovarian, pancreas, liver), chronic hepatitis B, pulmonary tuberculosis, keloid, drug eruption, heat cramp
Cohort3	1957	BioBank Japan	Illumina OmniExpress	1.045	2,283,889	60.90+−9.47	Cancer (esophageal, uterus) brain aneurysm, chronic obstractive lung disease, glaucoma
Cohort4	1,143	BioBank Japan	Illumina HumanHap 550	1.008	2,220,799	37.86+−8.13	Endometriosis
Metaanalysis	15,495			1.039	2,310,762	57.56+−14.15	

**Figure 1:**
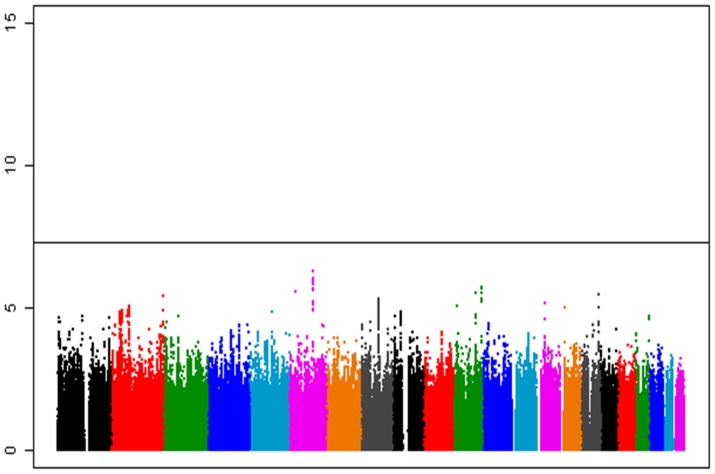
Results from meta-analysis of four genome-wide association studies. A total of 15,495 female samples were analyzed in this study.

**Figure 2:**
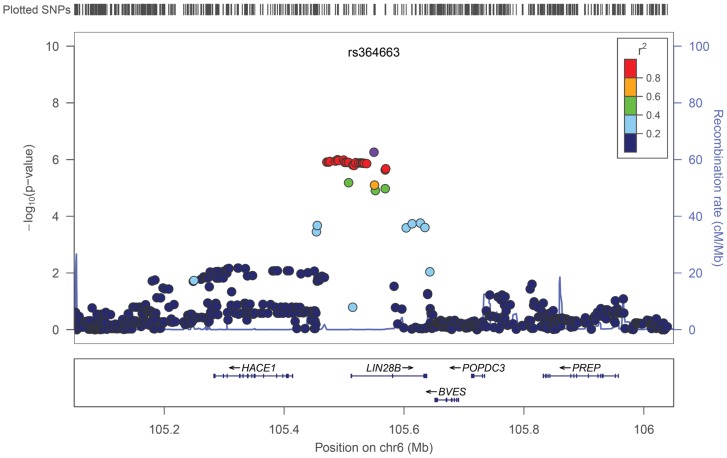
Regional association plot at rs364663. Upper panel; *P* values of genotyped SNPs are plotted (as −log_10_ values) against their physical location on chromosome 6 (NCBI Build 36). Estimated recombination rates from HapMap JPT shows the local LD structure. Inset; Colors of other SNPs indicate LD with rs2596542 according to a scale from *r* ^2^ = 0 to *r* ^2^ = 1 based on pair-wise *r* ^2^ values from HapMap JPT. Lower panel; Gene annotations from the University of California Santa Cruz genome browser.

The associations of these imputed SNPs with AAM were evaluated using a linear regression model [30] and meta-analysis. Quantile-Quantile plots of P-values indicated the Inflation factors to be as low as 1.039 (**Figure 1**), suggesting no substantial population stratification existed in our study population. Although we could not identify significant association that satisfied the genome-wide significant threshold (P<5×10−8), SNP rs364663 which is located within intron 2 of the *LIN28B* gene at 6q21 indicated the strongest association with a P-value of 5.49×10^−7^ (**Table 2**, **Figure 2**). SNP rs364663 exhibited the association with AAM in all four cohorts without significant heterogeneity in both effect sizes and directions (P-value for heterogeneity = 0.29; **Table 3**), suggesting that the observed associations at *LIN28B* are not the result of false-positives due to study-specific bias. Regional p-value plots indicated that all of the AMM-associated SNPs were clustered around the *LIN28B* locus (**Figure 2**). Previously reported SNPs rs314263 and rs7759938 near the *LIN28B* locus 21,24 also associated with AAM (P = 1.03×10−6 and 1.12×10−6, respectively) in the same direction. In addition to the 6q21 locus, 17 SNPs in seven genomic regions exhibited suggestive association with a p-value of<1×10−5 (**Table 2**). To further investigate the physiological role of these loci, the associations of these variations with body mass index (BMI) and height were evaluated using previously published results in 26,620 Japanese subjects [31]. SNP rs9404590 near the *LIN28B* locus associated with height with p-value of 0.0003 (Table S2), but we did not find significant association (P<0.01) between these loci and BMI (Table S2).

**Table 2:** The result of genome wide association analysis of age at menarche.

									
SNP	Chr	Position	Gene	allele		Allele 1 freq.	Beta	SE	P
				1	2				
rs364663	6	105549882	*LIN28B*	A	T	0.71679	−0.0893	0.01784	5.49×10^−7^
rs12200251	6	105489108	*LIN28B*	A	G	0.71854	−0.0868	0.01775	1.00×10^−6^
rs314263	6	105499438	*LIN28B*	T	C	0.71852	−0.0867	0.01775	1.03×10^−6^
rs2095812	6	105490671	*LIN28B*	G	C	0.28544	0.08667	0.01777	1.08×10^−6^
rs7759938	6	105485647	*LIN28B*	T	C	0.71771	−0.0865	0.01777	1.12×10^−6^
rs4946651	6	105476203	*LIN28B*	A	G	0.27974	0.08708	0.0179	1.15×10^−6^
rs11156429	6	105471114	*LIN28B*	T	G	0.28116	0.08646	0.01782	1.22×10^−6^
rs9391253	6	105474309	*LIN28B*	A	T	0.71867	−0.0865	0.01782	1.22×10^−6^
rs314262	6	105501314	*LIN28B*	A	G	0.71838	−0.086	0.01775	1.26×10^−6^
rs314280	6	105507530	*LIN28B*	A	G	0.28161	0.086	0.01775	1.26×10^−6^
rs395962	6	105504111	*LIN28B*	T	G	0.28161	0.086	0.01775	1.26×10^−6^
rs314274	6	105519625	*LIN28B*	A	C	0.28305	0.08279	0.01709	1.28×10^−6^
rs1744206	6	105530624	*LIN28B*	G	C	0.28307	0.08271	0.01709	1.31×10^−6^
rs314266	6	105528010	*LIN28B*	T	C	0.71684	−0.0827	0.01709	1.31×10^−6^
rs314268	6	105524671	*LIN28B*	A	G	0.71677	−0.0827	0.01709	1.31×10^−6^
rs314290	6	105533687	*LIN28B*	A	G	0.283	0.08263	0.01709	1.34×10^−6^
rs314291	6	105531594	*LIN28B*	T	C	0.71699	−0.0826	0.01709	1.34×10^−6^
rs314289	6	105537627	*LIN28B*	T	C	0.71703	−0.0825	0.01709	1.39×10^−6^
rs314276	6	105514692	*LIN28B*	A	C	0.28276	0.08522	0.01775	1.57×10^−6^
rs167539	6	105516741	*LIN28B*	A	C	0.71714	−0.0851	0.01775	1.61×10^−6^
rs7114000	11	126112790	*KIRREL3*	A	G	0.06837	0.15025	0.03164	2.04×10^−6^
rs3862645	11	126113656	*KIRREL3*	A	G	0.06837	0.15019	0.03164	2.07×10^−6^
rs314270	6	105569569	*LIN28B*	T	C	0.2829	0.0843	0.01777	2.10×10^−6^
rs314272	6	105568697	*LIN28B*	A	G	0.71716	−0.084	0.01777	2.26×10^−6^
rs314273	6	105568575	*LIN28B*	T	G	0.28266	0.08396	0.01777	2.31×10^−6^
rs1106419	11	126114297	*KIRREL3*	A	G	0.06846	0.14829	0.0318	3.12×10^−6^
rs12800752	11	99911850	*ARHGAP42*	T	C	0.80147	0.09455	0.02028	3.12×10^−6^
rs310008	16	80031150	*CMIP*	G	C	0.13392	−0.1076	0.02322	3.57×10^−6^
rs6431393	2	236204046	*AGAP1*	A	G	0.4948	0.07757	0.01681	3.95×10^−6^
rs4735738	8	77774180	*ZFHX4*	A	G	0.49918	0.07283	0.01594	4.93×10^−6^
rs3862642	11	126110244	*KIRREL3*	T	C	0.06969	0.1438	0.03149	4.97×10^−6^
rs6995390	8	77773567	*ZFHX4*	A	T	0.49503	0.07271	0.01597	5.30×10^−6^
rs3889461	11	126113915	*KIRREL3*	T	C	0.93023	−0.1426	0.03149	5.98×10^−6^
rs9404590	6	105507706	*LIN28B*	T	G	0.77319	−0.0847	0.01879	6.49×10^−6^
rs7822501	8	77825351	*ZFHX4*	A	G	0.51105	0.06865	0.01529	7.16×10^−6^
rs7822914	8	77825593	*ZFHX4*	T	C	0.48895	−0.0687	0.01529	7.16×10^−6^
rs6472982	8	77825957	*ZFHX4*	T	C	0.48894	−0.0685	0.01529	7.44×10^−6^
rs2076751	14	36059170	*NKX2-1*	A	C	0.23377	−0.087	0.01941	7.45×10^−6^
rs6472983	8	77834432	*ZFHX4*	T	G	0.48902	−0.0685	0.01529	7.56×10^−6^
rs369065	6	105550751	*LIN28B*	T	C	0.65066	−0.0721	0.01615	7.92×10^−6^
rs1865294	8	77772597	*ZFHX4*	A	G	0.50188	0.07121	0.01599	8.41×10^−6^
rs7114467	11	15414043	*INSC*	A	G	0.45957	−0.0713	0.01601	8.52×10^−6^

Genotyping result of 15,495 Japanese subjects were anlayzed in this study. Imputed SNPs with R2 of less than 0.7 were excluded from this analysis. A1 frequency of JPT was those from release 24 Hapmap JPT.

* Effect size and SE of allele1 on age at menarche (year per allele) and P-values were obtained by inverse-variance method.

**Table 3:** Association of rs364663 with age at menarche.

Groups	Chr	allele		Number of samples	Allele 1 Freq.	Beta*	SE*	P
	Position	1	2					
Cohort1		A	T	11454	0.72	−0.069	0.021	7.71×10^−4^
Cohort2	6			941	0.71	−0.131	0.068	0.056
Cohort3				1957	0.72	−0.137	0.050	0.0064
Cohort4	105549882			1143	0.71	−0.163	0.060	0.0071
Metaanalysis *				15495	0.72	−0.089	0.018	5.49×10^−7^

* Effect size and S.E. of allele1 on age at menarche (year per allele) and P-values were obtained by inverse-variance method.

Since AAM is associated with various disease risks, we conducted separate analyses for each disease and then performed meta-analysis for the top 42 loci. As a result, some SNPs showed slightly stronger association, but none cleared the genome wide significant threshold (Table S3). Similar to the current study, our group had previously conducted QTL analyses using disease status as a covariate and successfully identified many QTL loci [31]–[35]. Therefore, different background due to disease status was unlikely to significantly affect the result of association analysis.

Additionally, we examined the loci already known to show significant association with AAM in women of European ancestry [22]–[25], [36]. We selected 37 SNPs for this candidate analysis and successfully obtained the genotyping results of 33 SNPs (**Table 4**), and the risk allele was consistent with previous reports for 31 SNPs. In addition, eight SNPs in or near *RXRG*, *CCDC85A*, *LOC100421670*, *TMEM38B*, *ZNF483*, *ARNTL*, and *CA10* indicated possible associations with AAM (P<0.05). Among them, rs4452860 and rs7028916 at the *TMEM38B* locus exhibited significant association even after Bonferroni's correction (P<0.0015 = 0.05/33). Taken together, these variations as well as *LIN28B* are likely to be common AAM loci, although their effect sizes are different between women of European ancestry and those of Japanese.

**Table 4:** Association results in Japanese woman of previously identified SNPs with age at menarche in Caucasian woman.

SNP	Chr	Position	Gene	allele		Allele 1 freq.	Beta*	S.E.*	P	ref	concordance**
				1	2						
rs466639	1	163,661,506	*RXRG*	T	C	0.204	−0.0431	0.0196	0.028	1)	yes
rs633715	1	176,119,203	*SEC16B*	T	C	0.779	0.0312	0.0188	0.097	1)	yes
rs2947411	2	604,168	*TMEM18*	A	G	0.096	0.0519	0.0268	0.053	1)	yes
rs17268785	2	56,445,587	*CCDC85A*	A	G	0.793	−0.0561	0.0196	0.004	1)	yes
rs17188434	2	156,805,022	*NR4A2*	N.D.						1)	N.D.
rs12617311	2	199,340,810	*PLCL1*	A	G	0.432	−0.0026	0.0174	0.883	1)	yes
rs7617480	3	49,185,736	*KLHDC8B*	N.D.						1)	N.D.
rs6762477	3	50,068,213	*RBM6*	A	G	0.858	0.0219	0.0250	0.380	1)	yes
rs7642134	3	86,999,572	*VGLL3*	A	G	0.463	−0.0167	0.0173	0.334	1)	yes
rs6438424	3	119,057,512	*LOC100421670*	A	C	0.370	−0.0455	0.0162	0.005	1)	yes
rs6439371	3	134,093,442	*TMEM108*, *NPHP3*	A	G	0.705	−0.0080	0.0178	0.655	1)	yes
rs2002675	3	187,112,262	*TRA2B*, *ETV5*	A	G	0.862	−0.0128	0.0231	0.581	1)	yes
rs13187289	5	133,877,076	*PHF15*	G	C	0.078	0.0440	0.0295	0.137	1)	yes
rs13357391	5	136,468,981	*SPOCK*	T	C	0.840	−0.0248	0.0221	0.262	3)	yes
rs1859345	5	136,475,319	*SPOCK*	T	C	0.838	−0.0213	0.0221	0.336	3)	yes
rs4840086	6	100,315,159	*PRDM13, MCHR2*	A	G	0.619	−0.0251	0.0161	0.118	1)	no
rs1361108	6	126,809,293	*C6orf173, TRMT11*	N.D.						1)	N.D.
rs1079866	7	41,436,618	*INHBA*	G	C	0.298	0.0150	0.0170	0.376	1)	yes
rs7821178	8	78,256,392	*PXMP3*	A	C	0.436	−0.0150	0.0170	0.380	1)	yes
rs4452860	9	107,965,210	*TMEM38B*	A	G	0.545	0.0513	0.0160	0.0013	2)	yes
rs7028916	9	107,966,889	*TMEM38B*	A	C	0.454	−0.0512	0.0160	0.0013	2)	yes
rs7861820	9	107,976,495	*TMEM38B*	T	C	0.204	0.0363	0.0196	0.064	2)	yes
rs2090409	9	108,006,909	*TMEM38B*	N.D.						1)	N.D.
rs12684013	9	108,037,935	*TMEM38B*	T	C	0.525	−0.0265	0.0160	0.099	2)	yes
rs10980926	9	113,333,455	*ZNF483*	A	G	0.633	0.0441	0.0161	0.006	1)	yes
rs4929923	11	8,595,776	*TRIM66*	T	C	0.624	0.0068	0.0161	0.674	1)	yes
rs900145	11	13,250,481	*ARNTL*	T	C	0.489	−0.0371	0.0160	0.020	1)	yes
rs10899489	11	77,773,021	*GAB2*	A	C	0.439	0.0257	0.0160	0.109	1)	yes
rs6589964	11	122,375,893	*BSX*	A	C	0.549	−0.0137	0.0183	0.453	1)	yes
rs6575793	14	100,101,970	*BEGAIN*	T	C	0.328	−0.0279	0.0179	0.119	1)	yes
rs1659127	16	14,295,806	*MKL2*	A	G	0.483	0.0085	0.0161	0.595	1)	yes
rs9939609	16	52,378,028	*FTO*	A	T	0.198	−0.0191	0.0196	0.328	1)	yes
rs1364063	16	68,146,073	*NFAT5*	T	C	0.864	−0.0236	0.0231	0.307	1)	no
rs9635759	17	46,968,784	*CA10*	A	G	0.435	0.0479	0.0172	0.005	1)	yes
rs1398217	18	43,006,236	*FUSSEL18*	G	C	0.599	−0.0225	0.0161	0.161	1)	yes
rs10423674	19	18,678,903	*CRTC1*	A	C	0.705	0.0031	0.0171	0.856	1)	yes
rs852069	20	17,070,593	*PCSK2*	A	G	0.776	−0.0340	0.0187	0.069	1)	yes

Genotyping result of 15,495 Japanese subjects were anlayzed in this study. Imputed SNPs with R2 of less than 0.7 were excluded from this analysis. A1 frequency of JPT were those from release 24 Hapmap JPT. N.D.; no data. References: 1 Elks et al Nat Genet 2010, 2 He et al Nature Genet 2009, 3 Liu et al Plos Genet 2009. P-value of 0.0015 (0.05/33) was set at the significant threshold for this candidate analysis.

* :Effect size and S.E. of allele1 on age at menarche (year per allele) and P-values were obtained by inverse-variance method.

** Concordance of association direction between this study and the previous report.

## Discussion

AAM is a complex trait that is influenced by both genetic and environmental factors. In recent years, genome wide association analyses have become a standard method to identify genetic factors related with various diseases and phenotypes. In 2002 our group performed the first GWAS for myocardial infarction and successfully identified *LTA* as a disease susceptibility gene. Using this method, we have identified a number of loci associated with various phenotypes and common diseases [37]–[42].

Through the analysis of more than 15,000 Japanese female subjects from the Biobank Japan Project, we here report the association of *LIN28B* with AAM. In our analysis, SNP rs364663 within intron 2 of the *LIN28B* gene exhibited the strongest association. Previously reported SNPs rs314263, rs7759938, rs314280, and rs314276 near or in the *LIN28B* gene also showed equivalent association with p-values of 1.03−1.57×10−6. Although the directions of associations were consistent between women of European ancestry and those of Japanese, the effect sizes among Japanese (0.085–0.087) were less than those among European ancestry (0.09–0.14). Thus *LIN28B* is a common AAM loci, but its effects are relatively small among the Japanese population.

The lin-28 gene was originally identified through C. elegans mutants showing abnormality in developmental timing [43]. Deleterious mutations in lin-28 produce an abnormal rapid tempo of development through larval stages to adult cuticle formation [43]. Two mammalian homologs, Lin28a and Lin28b, possess similar biochemical activities [44], [45]. Transgenic mice expressing Lin28a exhibited increased body size, crown-rump length and delayed onset of puberty due to increased glucose metabolism and insulin sensitivity [46]. In humans, both *LIN28B* and *LIN28A* control preprocessing of the let7 microRNA family [45]. *Lin28B* is highly expressed in the majority of human hepatocellular carcinomas and embryonic stem cells [47], and regulate cell pluripotency [48] as well as cancer growth [47]. However the role of genetic variations in the *LIN28B* locus should be investigated in the future study.

Among the seven suggestive loci indentified in our GWAS, *ZFHX4* and *NKX2.1* loci were previously shown to be associated with height in the Caucasian population [49], [50]. These loci also exhibited association with height among the Japanese population (p = 0.053 and 0.023, respectively). The *NKX2.1* gene encodes a thyroid-specific transcription factor that was shown to be associated with hypothyroidism [51]. Since delayed pubertal development is a common manifestation of hypothyroidism, variation at *NKX2.1* locus might affect AAM through the regulation of serum thyroid hormone level. The *ZFHX4* gene encodes a homeodomain-zinc finger protein. *ZFHX4* mRNA is highly expressed in brain, liver, and muscle, however the molecular mechanism whereby variations within this genetic locus affect AAM is not clear. In addition, *KIRREL3* locus was shown to be associated with breast size in women of European ancestry [52]. The *KIRREL3* gene encode a member of the nephrin-like protein family. *KIRREL3* was highly expressed in brain and kidney, and shown to be associated with mental retardation autosomal dominant type 4 [53] and neurocognitive delay associated with Jacobsen Syndrome [54]. Taken together, these three loci seem to be common development traits in women of both European ancestry and Japanese.

In our candidate analysis, the association of SNPs rs4452860 and rs7028916 on 9q31.2 with AAM was also validated. This locus was repeatedly shown to be associated with AAM [25], [55]as well as serum ALT (alanine transaminase) level[56]. SNPs rs4452860 and rs7028916 were located more than 300 kb away from the *TMEM38B* gene. In mice, *TMEM38B* is strongly expressed in brain, and *TMEM38B* deficient mice is neonatal lethal[57]. In humans, a homozygous mutation of *TMEM38B* was associated with autosomal recessive osteosgenesis imperfecta[58]. These findings also suggest an important role of TMEM38B in developmental processes.

In this study, we observed early AAM among breast cancer patients compared with overall samples (12.99 vs 13.44, **Table S1**). On the other hand, patients with osteoporosis exhibited late AAM (14.33 y.o.). These findings are concordant with previous reports. Since we do not have age-matched controls, the association of these diseases and AAM should be examined using more appropriate sample sets.

Although more than 15,000 samples were employed in this study, no SNP cleared the genome wide significance threshold (p<5×10^−8^). This could be explained by some limitations in our study. Firstly, the information about AAM was self-reported and might not be very accurate. Although this distinct event is often well recalled many years later [59], possible error due to age-associated memory impairment would increase the risk of false negative association. Secondly, we did not have a replication sample set, and the total sample size may be inadequate to detect variations with modest effects. At present, we unfortunately do not have sufficient samples for replication, since more than 2,900 additional samples are necessary to obtain a genome wide significant association for the top SNP rs364663. Previous studies comprising up to 17,510 women detected only one or two genome-wide significant signals [22]–[25]. However, 30 significant loci were identified by using 87,802 subjects in the screening stage [21]. Although no SNPs reached the genome wide significance in our study, analyzing an increased number of subjects in a relatively younger generation would improve statistical power as well as data accuracy, and subsequently enable us to identify other genetic factors with modest effects.

## Methods

### Samples

The subjects enrolled in the GWAS meta-analysis for age at menarche (AAM) (n = 15,495) consisted of female patients that were classified into 33 disease groups; cancer (lung, esophagus, stomach, colon, liver, cholangiocarcinoma, pancreas, breast, uterine cervix, uterine body, ovary, hematopoietic organ), diabetes, myocardial infarction, brain infarction, arteriosclerosis, arrhythmia, drug eruption, liver cirrhosis, amyotrophic lateral sclerosis, osteoporosis, fibroid, and drug response, rheumatoid arthritis. chronic hepatitis B, pulmonary tuberculosis, keloid, heat cramp, brain aneurysm, chronic obstractive lung disease, glaucoma, and endometriosis (**Tables 1** **and** **2**). All subjects were collected under the support of the BioBank Japan Project [26], in which the individuals with any one of the 47 common diseases were enrolled between 2003 and 2008. Subjects under 17 years of age were not included in this study. Various life style information such as AAM was obtained through a face-to-face interview by trained medical coordinators using questionnaire sheets at each hospital. All participants provided written informed consent as approved by the ethical committees of each institute. For participants between the age of 17 and 20 years old, we obtained written informed consent from both the participant and her parents. AAM was collected by self-report on the questionnaire. The subjects with AAM between the ages of 10 and 17 were enrolled for the study. This project was approved by the ethical committees of the BioBank Japan Project [26] and the University of Tokyo.

### Genotyping and quality control

In the four GWAS enrolled in the meta-analysis of AAM, all samples were genotyped at more than 500,000 loci using one of the following platforms: Illumina HumanHap550v3 Genotyping BeadChip, Illumina HumanHap610-Quad Genotyping BeadChip, or Illumina Omni express Genotyping BeadChip (**Table 1**). Then we applied the following quality control for each GWAS separately: exclusion criteria; subjects with call rates<0.98, SNPs with call rates<0.99 or with ambiguous clustering of the intensity plots, or non-autosomal SNPs. We then excluded subjects whose ancestries were estimated to be distinct from East-Asian populations using principle component analysis performed by EIGENSTRAT version 2.0 [27]. Subsequently, the SNPs with MAF<0.01 or P-value of the Hardy-Weinberg equilibrium test<1.0×10^−7^ were excluded.

After the quality control criteria mentioned above were applied, genotype imputation was performed by MACH 1.0.16 [29] using the genotype data of Phase II HapMap JPT and CHB individuals (release 24)[28] as references, in a two-step procedure as described elsewhere [60]. In the first step of the imputation, recombination and error rate maps were estimated using 500 subjects randomly selected from the GWAS data. In the second step, imputation of the genotypes of all subjects was performed using the rate maps estimated in the first step. Quality control filters of MAF≥0.01 and *Rsq* values≥0.7 were applied for the imputed SNPs.

### Statistical analysis

Associations of the SNPs with AAM were assessed by linear regression assuming the additive effects of the allele dosages on AAM, using mach2qtl software [29]. Years of birth and affection statuses of the diseases were used as covariates. Meta-analysis of all four GWAS was performed using an inverse-variance method from the summary statistics of beta and standard error (SE), using the Java source code implemented by the authors [61]. Genomic control correction was applied for each GWAS separately, and applied again for the results of GWAS meta-analysis. We set the P*-*values of 5.0×10^−8^ and 0.0015 ( = 0.05/33, Bonferroni's correction based on the numbers of the evaluated loci) as significance thresholds in GWAS and candidate gene analysis, respectively. Heterogeneity of the effect sizes among the studies was evaluated using Cochran's Q statistics.

### Body mass index and Height QTL analysis

The associations of 42 SNPs with BMI and height were evaluated using previously published results, in which a total of 26,620 subjects with 32 diseases from Biobank Japan were enrolled [31]. Of the 26,620 subjects, 12,350 were also included in this AAM study. Genotyping was performed using the Illumina HumanHap610-Quad Genotyping BeadChip. Genotype imputation was performed using MACH 1.0. BMI was calculated based on self-reported body weight and height data. A rank-based inverse-normal transformation was applied to the BMI values of the subjects. Associations of the SNPs with transformed values of BMI were assessed by linear regression assuming additive effects of allele dosages (bound between 0.0 and 2.0) using mach2qtl software using gender, age, smoking history, the affection statuses of the diseases and the demographic classifications of the medical institutes in Japan where the subjects were enrolled were used as covariates.

### Web resources

The URLs for data presented herein are as follows. BioBank Japan Project, http://biobankjp.org

MACH and mach2qtl software, http://www.sph.umich.edu/csg/abecasis/MACH/index.html International HapMap Project, http://www.hapmap.org PLINK software, http://pngu.mgh.harvard.edu/~purcell/plink/index.shtml EIGENSTRAT software, http://genepath.med.harvard.edu/~reich/Software.htm *R* statistical software, http://cran.r-project.org.

## Supporting Information

Table S1 **Age at menarche in each disease cohort.**
(DOCX)

Table S2 **The result of association analysis of TOP 42 SNPs from Japanese AAM study with body mass index and height from previous GWAS by Okada et al.**
(DOCX)

Table S3 **The result of top 42 SNPs by separated analysis.**
(DOCX)

## References

[1] Tena-Sempere M (2006) GPR54 and kisspeptin in reproduction. Hum Reprod Update 12: 631–639. 16731583 10.1093/humupd/dml023

[2] Susman EJ, Nottelmann ED, Inoff-Germain GE, Dorn LD, Cutler GB Jr, et al (1985) The relation of relative hormonal levels and physical development and social-emotional behavior in young adolescents. Journal of Youth and Adolescence 14: 245–264. 24301179 10.1007/BF02090322

[3] Kaltiala-Heino R, Kosunen E, Rimpela M (2003) Pubertal timing, sexual behaviour and self-reported depression in middle adolescence. J Adolesc 26: 531–545. 12972267 10.1016/s0140-1971(03)00053-8

[4] Kaltiala-Heino R, Rimpela M, Rissanen A, Rantanen P (2001) Early puberty and early sexual activity are associated with bulimic-type eating pathology in middle adolescence. J Adolesc Health 28: 346–352. 11287254 10.1016/s1054-139x(01)00195-1

[5] Freedman DS, Khan LK, Serdula MK, Dietz WH, Srinivasan SR, et al (2003) The relation of menarcheal age to obesity in childhood and adulthood: the Bogalusa heart study. BMC pediatrics 3: 3. 12723990 10.1186/1471-2431-3-3 PMC156622

[6] Kjaer K, Hagen C, Sandø S, Eshøj O (1992) Epidemiology of menarche and menstrual disturbances in an unselected group of women with insulin-dependent diabetes mellitus compared to controls. Journal of Clinical Endocrinology & Metabolism 75: 524–529. 1639955 10.1210/jcem.75.2.1639955

[7] Velie EM, Nechuta S, Osuch JR (2005) Lifetime reproductive and anthropometric risk factors for breast cancer in postmenopausal women. Breast Dis 24: 17–35. 16917137 10.3233/bd-2006-24103

[8] Cooper GS, Ephross SA, Weinberg CR, Baird DD, Whelan EA, et al (1999) Menstrual and reproductive risk factors for ischemic heart disease. Epidemiology 10: 255–259. 10230834

[9] Fujiwara S, Kasagi F, Yamada M, Kodama K (1997) Risk factors for hip fracture in a Japanese cohort. Journal of Bone and Mineral Research 12: 998–1004. 9199997 10.1359/jbmr.1997.12.7.998

[10] Onland-Moret N, Peeters P, Van Gils C, Clavel-Chapelon F, Key T, et al (2005) Age at menarche in relation to adult height. Am J Epidemiol 162: 623–632. 16107566 10.1093/aje/kwi260

[11] Anderson CA, Duffy DL, Martin NG, Visscher PM (2007) Estimation of variance components for age at menarche in twin families. Behav Genet 37: 668–677. 17680356 10.1007/s10519-007-9163-2

[12] Towne B, Czerwinski SA, Demerath EW, Blangero J, Roche AF, et al (2005) Heritability of age at menarche in girls from the Fels Longitudinal Study. American journal of physical anthropology 128: 210–219. 15779076 10.1002/ajpa.20106

[13] Treloar SA, Martin NG (1990) Age at menarche as a fitness trait: nonadditive genetic variance detected in a large twin sample. Am J Hum Genet 47: 137–148. 2349942 PMC1683767

[14] Anderson CA, Zhu G, Falchi M, Van Den Berg SM, Treloar SA, et al (2008) A genome-wide linkage scan for age at menarche in three populations of European descent. Journal of Clinical Endocrinology & Metabolism 93: 3965. 18647812 10.1210/jc.2007-2568 PMC2579643

[15] Stavrou I, Zois C, Ioannidis JP, Tsatsoulis A (2002) Association of polymorphisms of the oestrogen receptor alpha gene with the age of menarche. Hum Reprod 17: 1101–1105. 11925413 10.1093/humrep/17.4.1101

[16] Stavrou I, Zois C, Chatzikyriakidou A, Georgiou I, Tsatsoulis A (2006) Combined estrogen receptor alpha and estrogen receptor beta genotypes influence the age of menarche. Hum Reprod 21: 554–557. 16210384 10.1093/humrep/dei326

[17] Guo Y, Xiong DH, Yang TL, Guo YF, Recker RR, et al (2006) Polymorphisms of estrogen-biosynthesis genes CYP17 and CYP19 may influence age at menarche: a genetic association study in Caucasian females. Hum Mol Genet 15: 2401–2408. 16782804 10.1093/hmg/ddl155 PMC1803760

[18] Xita N, Tsatsoulis A, Stavrou I, Georgiou I (2005) Association of SHBG gene polymorphism with menarche. Mol Hum Reprod 11: 459–462. 15879463 10.1093/molehr/gah178

[19] Rothenbuhler A, Fradin D, Heath S, Lefevre H, Bouvattier C, et al (2006) Weight-adjusted genome scan analysis for mapping quantitative trait Loci for menarchal age. The Journal of clinical endocrinology and metabolism 91: 3534–3537. 16804047 10.1210/jc.2006-0150

[20] Guo Y, Shen H, Xiao P, Xiong DH, Yang TL, et al (2006) Genomewide linkage scan for quantitative trait loci underlying variation in age at menarche. The Journal of clinical endocrinology and metabolism 91: 1009–1014. 16394082 10.1210/jc.2005-2179

[21] Elks CE, Perry JR, Sulem P, Chasman DI, Franceschini N, et al (2010) Thirty new loci for age at menarche identified by a meta-analysis of genome-wide association studies. Nat Genet 42: 1077–1085. 21102462 10.1038/ng.714 PMC3140055

[22] Sulem P, Gudbjartsson DF, Rafnar T, Holm H, Olafsdottir EJ, et al (2009) Genome-wide association study identifies sequence variants on 6q21 associated with age at menarche. Nat Genet 41: 734–738. 19448622 10.1038/ng.383

[23] Ong KK, Elks CE, Li S, Zhao JH, Luan J, et al (2009) Genetic variation in LIN28B is associated with the timing of puberty. Nature genetics 41: 729–733. 19448623 10.1038/ng.382 PMC3000552

[24] He C, Kraft P, Chen C, Buring JE, Pare G, et al (2009) Genome-wide association studies identify loci associated with age at menarche and age at natural menopause. Nature genetics 41: 724–728. 19448621 10.1038/ng.385 PMC2888798

[25] Perry JR, Stolk L, Franceschini N, Lunetta KL, Zhai G, et al (2009) Meta-analysis of genome-wide association data identifies two loci influencing age at menarche. Nat Genet 41: 648–650. 19448620 10.1038/ng.386 PMC2942986

[26] Nakamura Y (2007) The BioBank Japan Project. Clin Adv Hematol Oncol 5: 696–697. 17982410

[27] Price AL, Patterson NJ, Plenge RM, Weinblatt ME, Shadick NA, et al (2006) Principal components analysis corrects for stratification in genome-wide association studies. Nat Genet 38: 904–909. 16862161 10.1038/ng1847

[28] The International HapMap Consortium (2003) The International HapMap Project. Nature 426: 789–796. 14685227 10.1038/nature02168

[29] Li Y, Willer C, Sanna S, Abecasis G (2009) Genotype imputation. Annu Rev Genomics Hum Genet 10: 387–406. 19715440 10.1146/annurev.genom.9.081307.164242 PMC2925172

[30] Li J, Guo YF, Pei Y, Deng HW (2012) The impact of imputation on meta-analysis of genome-wide association studies. PLoS One 7: e34486. 22496814 10.1371/journal.pone.0034486 PMC3320624

[31] Okada Y, Kubo M, Ohmiya H, Takahashi A, Kumasaka N, et al (2012) Common variants at CDKAL1 and KLF9 are associated with body mass index in east Asian populations. Nat Genet 44: 302–306. 22344221 10.1038/ng.1086 PMC3838874

[32] Okada Y, Sim X, Go MJ, Wu JY, Gu D, et al (2012) Meta-analysis identifies multiple loci associated with kidney function-related traits in east Asian populations. Nat Genet 44: 904–909. 22797727 10.1038/ng.2352 PMC4737645

[33] Okada Y, Hirota T, Kamatani Y, Takahashi A, Ohmiya H, et al (2011) Identification of nine novel loci associated with white blood cell subtypes in a Japanese population. PLoS Genet 7: e1002067. 21738478 10.1371/journal.pgen.1002067 PMC3128095

[34] Okada Y, Kamatani Y, Takahashi A, Matsuda K, Hosono N, et al (2010) A genome-wide association study in 19 633 Japanese subjects identified LHX3-QSOX2 and IGF1 as adult height loci. Hum Mol Genet 19: 2303–2312. 20189936 10.1093/hmg/ddq091

[35] Okada Y, Kamatani Y, Takahashi A, Matsuda K, Hosono N, et al (2010) Common variations in PSMD3-CSF3 and PLCB4 are associated with neutrophil count. Hum Mol Genet 19: 2079–2085. 20172861 10.1093/hmg/ddq080

[36] He C, Kraft P, Chasman DI, Buring JE, Chen C, et al (2010) A large-scale candidate gene association study of age at menarche and age at natural menopause. Human genetics 128: 515–527. 20734064 10.1007/s00439-010-0878-4 PMC2967297

[37] Kumar V, Kato N, Urabe Y, Takahashi A, Muroyama R, et al (2011) Genome-wide association study identifies a susceptibility locus for HCV-induced hepatocellular carcinoma. Nat Genet In press 10.1038/ng.809 21499248

[38] Kumar V, Kato N, Urabe Y, Takahashi A, Muroyama R, et al (2011) Genome-wide association study identifies a susceptibility locus for HCV-induced hepatocellular carcinoma. Nat Genet 43: 455–458. 21499248 10.1038/ng.809

[39] Tanikawa C, Urabe Y, Matsuo K, Kubo M, Takahashi A, et al (2012) A genome-wide association study identifies two susceptibility loci for duodenal ulcer in the Japanese population. Nat Genet In press 10.1038/ng.1109 22387998

[40] Mbarek H, Ochi H, Urabe Y, Kumar V, Kubo M, et al (2011) A genome-wide association study of chronic hepatitis B identified novel risk locus in a Japanese population. Hum Mol Genet 20: 3884–3892. 21750111 10.1093/hmg/ddr301

[41] Urabe Y, Tanikawa C, Takahashi A, Okada Y, Morizono T, et al (2012) A Genome-Wide Association Study of Nephrolithiasis in the Japanese Population Identifies Novel Susceptible Loci at 5q35. 3, 7p14. 3, and 13q14. 1. PLoS Genet 8: e1002541. 22396660 10.1371/journal.pgen.1002541 PMC3291538

[42] Cui R, Okada Y, Jang SG, Ku JL, Park JG, et al (2011) Common variant in 6q26–q27 is associated with distal colon cancer in an Asian population. Gut 60: 799–805. 21242260 10.1136/gut.2010.215947 PMC3095478

[43] Ambros V, Horvitz HR (1984) Heterochronic mutants of the nematode Caenorhabditis elegans. Science 226: 409–416. 6494891 10.1126/science.6494891

[44] Heo I, Joo C, Cho J, Ha M, Han J, et al (2008) Lin28 mediates the terminal uridylation of let-7 precursor MicroRNA. Molecular cell 32: 276–284. 18951094 10.1016/j.molcel.2008.09.014

[45] Viswanathan SR, Daley GQ, Gregory RI (2008) Selective blockade of microRNA processing by Lin28. Science's STKE 320: 97. 10.1126/science.1154040 PMC3368499 18292307

[46] Zhu H, Shah S, Shyh-Chang N, Shinoda G, Einhorn WS, et al (2010) Lin28a transgenic mice manifest size and puberty phenotypes identified in human genetic association studies. Nat Genet 42: 626–630. 20512147 10.1038/ng.593 PMC3069638

[47] Guo Y, Chen Y, Ito H, Watanabe A, Ge X, et al (2006) Identification and characterization of lin-28 homolog B (LIN28B) in human hepatocellular carcinoma. Gene 384: 51–61. 16971064 10.1016/j.gene.2006.07.011

[48] Yu J, Vodyanik MA, Smuga-Otto K, Antosiewicz-Bourget J, Frane JL, et al (2007) Induced pluripotent stem cell lines derived from human somatic cells. Science 318: 1917–1920. 18029452 10.1126/science.1151526

[49] Gudbjartsson DF, Walters GB, Thorleifsson G, Stefansson H, Halldorsson BV, et al (2008) Many sequence variants affecting diversity of adult human height. Nat Genet 40: 609–615. 18391951 10.1038/ng.122

[50] Lettre G, Jackson AU, Gieger C, Schumacher FR, Berndt SI, et al (2008) Identification of ten loci associated with height highlights new biological pathways in human growth. Nat Genet 40: 584–591. 18391950 10.1038/ng.125 PMC2687076

[51] Krude H, Schutz B, Biebermann H, Von Moers A, Schnabel D, et al (2002) Choreoathetosis, hypothyroidism, and pulmonary alterations due to human NKX2-1 haploinsufficiency. Journal of Clinical Investigation 109: 475–480. 11854319 10.1172/JCI14341 PMC150790

[52] Eriksson N, Benton GM, Do CB, Kiefer AK, Mountain JL, et al (2012) Genetic variants associated with breast size also influence breast cancer risk. BMC Med Genet 13: 53. 22747683 10.1186/1471-2350-13-53 PMC3483246

[53] Bhalla K, Luo Y, Buchan T, Beachem MA, Guzauskas GF, et al (2008) Alterations in CDH15 and KIRREL3 in patients with mild to severe intellectual disability. Am J Hum Genet 83: 703–713. 19012874 10.1016/j.ajhg.2008.10.020 PMC2668064

[54] Guerin A, Stavropoulos DJ, Diab Y, Chénier S, Christensen H, et al.. (2012) Interstitial deletion of 11q-implicating the KIRREL3 gene in the neurocognitive delay associated with Jacobsen syndrome. American Journal of Medical Genetics Part A. 10.1002/ajmg.a.35621 22965935

[55] Elks CE, Perry JR, Sulem P, Chasman DI, Franceschini N, et al (2010) Thirty new loci for age at menarche identified by a meta-analysis of genome-wide association studies. Nat Genet 42: 1077–1085. 21102462 10.1038/ng.714 PMC3140055

[56] Melzer D, Perry JR, Hernandez D, Corsi AM, Stevens K, et al (2008) A genome-wide association study identifies protein quantitative trait loci (pQTLs). PLoS Genet 4: e1000072. 18464913 10.1371/journal.pgen.1000072 PMC2362067

[57] Yazawa M, Ferrante C, Feng J, Mio K, Ogura T, et al (2007) TRIC channels are essential for Ca2+ handling in intracellular stores. Nature 448: 78–82. 17611541 10.1038/nature05928

[58] Volodarsky M, Markus B, Cohen I, Staretz-Chacham O, Flusser H, et al (2013) A Deletion Mutation in TMEM38B Associated with Autosomal Recessive Osteogenesis Imperfecta. Hum Mutat 10.1002/humu.22274 23316006

[59] Parent AS, Teilmann G, Juul A, Skakkebaek NE, Toppari J, et al (2003) The timing of normal puberty and the age limits of sexual precocity: variations around the world, secular trends, and changes after migration. Endocr Rev 24: 668–693. 14570750 10.1210/er.2002-0019

[60] Okada Y, Takahashi A, Ohmiya H, Kumasaka N, Kamatani Y, et al (2011) Genome-wide association study for C-reactive protein levels identified pleiotropic associations in the IL6 locus. Hum Mol Genet 20: 1224–1231. 21196492 10.1093/hmg/ddq551

[61] Yamaguchi-Kabata Y, Nakazono K, Takahashi A, Saito S, Hosono N, et al (2008) Japanese population structure, based on SNP genotypes from 7003 individuals compared to other ethnic groups: effects on population-based association studies. The American Journal of Human Genetics 83: 445–456. 18817904 10.1016/j.ajhg.2008.08.019 PMC2561928

