# Correction: Genome Wide Association Study of Age at Menarche in the Japanese Population

**DOI:** 10.1371/annotation/1238450b-9ba4-4afb-aee3-a3c11b4f5ea1

**Published:** 2013-10-08

**Authors:** Chizu Tanikawa, Yukinori Okada, Atsushi Takahashi, Katsutoshi Oda, Naoyuki Kamatani, Michiaki Kubo, Yusuke Nakamura, Koichi Matsuda

The affiliation of the editor, Chunyan He, is incorrect. The correct affiliation is: Richard M. Fairbanks School of Public Health, Indiana University, USA. 

