# Supplementary material for: Genome Wide Association Study of Age at Menarche in the Japanese Population
Source: PLoS One. 2013 May 7;8(5):e63821. doi: 10.1371/journal.pone.0063821 (PMC3646805; doi:10.1371/journal.pone.0063821)
Supplement: Supplementary file 1 [file pone.0063821.s001.docx]

fibroid	1521	12.34
rheumatic arthritis	1564	13.68
Warfarin response	307	13.93
drug adverse reaction	1540	13.49
drug eruption hyperactivation	38	13.59
**Cohort2**		
cervical cancer	171	12.44
uterus cancer	149	12.64
esophageal cancer	20	13.40
hematopoietic cancer	46	13.13
cholangiocarcinoma	44	14.16
ovarian cancer	120	12.73
pancreas cancer	35	13.91
liver cancer	1	13
chronic hepatitis B	61	12.72
pulmonary tuberculosis	34	13.18
keloid	108	12.45
drug eruption hyperactivation	190	13.18
heat cramp	1	12
**Cohort3**		
esophageal cancer	86	13.99
uterus cancer	566	13.40
brain aneurysm	685	13.76
chronic obstructive pulmonary disease	76	14.30
glaucoma	586	13.67
**Cohort4**		
endometriosis	1155	12.40
**Overall**	15495	13.44
