# Supplementary material for: Genome Wide Association Study of Age at Menarche in the Japanese Population
Source: PLoS One. 2013 May 7;8(5):e63821. doi: 10.1371/journal.pone.0063821 (PMC3646805; doi:10.1371/journal.pone.0063821)
Supplement: Supplementary file 2 [file pone.0063821.s002.docx]

Table S2 The result of association analysis of TOP 42 SNPs from Japanese AAM study with bc index and height from previous GWAS by Okada et al

SNP	Chr	Position	allele		BMI			Height	
			1	2	Beta*	S.E.*	P	Beta*	S.E.*
rs6431393	2	###	A	G	0.0191	0.0087	0.0274	0.0003	0.0060
rs11156429	6	###	G	T	0.0112	0.0090	0.2134	0.0208	0.0062
rs9391253	6	###	A	T	0.0112	0.0090	0.2134	0.0208	0.0062
rs4946651	6	###	A	G	-0.0112	0.0090	0.2137	-0.0209	0.0062
rs7759938	6	###	C	T	-0.0109	0.0090	0.2278	-0.0208	0.0062
rs12200251	6	###	A	G	0.0109	0.0090	0.2290	0.0208	0.0062
rs2095812	6	###	C	G	0.0107	0.0091	0.2388	0.0211	0.0062
rs314263	6	###	C	T	-0.0108	0.0090	0.2313	-0.0208	0.0062
rs314262	6	###	A	G	0.0106	0.0090	0.2423	0.0208	0.0062
rs395962	6	###	G	T	0.0106	0.0090	0.2424	0.0208	0.0062
rs314280	6	###	A	G	-0.0106	0.0090	0.2424	-0.0208	0.0062
rs9404590	6	###	G	T	-0.0225	0.0097	0.0207	-0.0240	0.0067
rs314276	6	###	A	C	-0.0102	0.0090	0.2607	-0.0207	0.0062
rs167539	6	###	A	C	0.0101	0.0090	0.2641	0.0207	0.0062
rs314274	6	###	A	C	-0.0100	0.0090	0.2694	-0.0207	0.0062
rs314268	6	###	A	G	0.0099	0.0090	0.2731	0.0207	0.0062
rs314266	6	###	C	T	-0.0099	0.0090	0.2742	-0.0207	0.0062
rs1744206	6	###	C	G	0.0098	0.0090	0.2761	0.0207	0.0062
rs314291	6	###	C	T	-0.0098	0.0090	0.2779	-0.0207	0.0062
rs314290	6	###	A	G	-0.0098	0.0090	0.2792	-0.0207	0.0062
rs314289	6	###	C	T	-0.0097	0.0090	0.2812	-0.0206	0.0062
rs364663	6	###	A	T	0.0107	0.0093	0.2467	0.0210	0.0064
rs369065	6	###	C	T	-0.0119	0.0086	0.1640	-0.0194	0.0059
rs314273	6	###	G	T	0.0097	0.0091	0.2862	0.0205	0.0062
rs314272	6	###	A	G	0.0097	0.0091	0.2882	0.0206	0.0062
rs314270	6	###	C	T	0.0096	0.0091	0.2890	0.0206	0.0062
rs1865294	8	77772597	A	G	-0.0179	0.0083	0.0323	0.0109	0.0057
rs6995390	8	77773567	A	T	-0.0143	0.0083	0.0828	0.0096	0.0057
rs4735738	8	77774180	A	G	-0.0150	0.0082	0.0690	0.0092	0.0057
rs7822501	8	77825351	A	G	-0.0151	0.0082	0.0648	0.0090	0.0056
rs7822914	8	77825593	C	T	-0.0150	0.0082	0.0651	0.0089	0.0056
rs6472982	8	77825957	C	T	-0.0150	0.0082	0.0651	0.0089	0.0056
rs6472983	8	77834432	G	T	-0.0151	0.0082	0.0646	0.0089	0.0056
rs7114467	11	15414043	A	G	0.0040	0.0082	0.6294	-0.0015	0.0057
rs12800752	11	99911850	C	T	0.0215	0.0104	0.0391	-0.0035	0.0072
rs3862642	11	###	C	T	0.0094	0.0161	0.5592	-0.0084	0.0110
rs7114000	11	###	A	G	-0.0089	0.0162	0.5826	0.0108	0.0111
rs3862645	11	###	A	G	-0.0088	0.0162	0.5875	0.0109	0.0111
rs3889461	11	###	C	T	-0.0078	0.0161	0.6280	0.0093	0.0110
rs1106419	11	###	A	G	-0.0088	0.0162	0.5854	0.0107	0.0111
