# Supplementary material for: Genome Wide Association Study of Age at Menarche in the Japanese Population
Source: PLoS One. 2013 May 7;8(5):e63821. doi: 10.1371/journal.pone.0063821 (PMC3646805; doi:10.1371/journal.pone.0063821)
Supplement: Supplementary file 3 [file pone.0063821.s003.docx]

rs3862645	11	126113656	*KIRREL3*	A	G	0.06837	0.1520	0.0303
rs314270	6	105569569	*LIN28B*	T	C	0.2829	0.0838	0.0167
rs314272	6	105568697	*LIN28B*	A	G	0.71716	-0.0836	0.0167
rs314273	6	105568575	*LIN28B*	T	G	0.28266	0.0835	0.0167
rs1106419	11	126114297	*KIRREL3*	A	G	0.06846	0.1500	0.0304
rs12800752	11	99911850	*ARHGAP42*	T	C	0.80147	0.0915	0.0191
rs310008	16	80031150	*CMIP*	G	C	0.13392	-0.1078	0.0224
rs6431393	2	236204046	*AGAP1*	A	G	0.4948	0.0778	0.0159
rs4735738	8	77774180	*ZFHX4*	A	G	0.49918	0.0705	0.0151
rs3862642	11	126110244	*KIRREL3*	T	C	0.06969	0.1463	0.0298
rs6995390	8	77773567	*ZFHX4*	A	T	0.49503	0.0705	0.0151
rs3889461	11	126113915	*KIRREL3*	T	C	0.93023	-0.1449	0.0298
rs9404590	6	105507706	*LIN28B*	T	G	0.77319	-0.0842	0.0180
rs7822501	8	77825351	*ZFHX4*	A	G	0.51105	0.0665	0.0149
rs7822914	8	77825593	*ZFHX4*	T	C	0.48895	-0.0665	0.0149
rs6472982	8	77825957	*ZFHX4*	T	C	0.48894	-0.0664	0.0149
rs2076751	14	36059170	*NKX2-1*	A	C	0.23377	-0.0847	0.0183
rs6472983	8	77834432	*ZFHX4*	T	G	0.48902	-0.0663	0.0149
rs369065	6	105550751	*LIN28B*	T	C	0.65066	-0.0710	0.0157
rs1865294	8	77772597	*ZFHX4*	A	G	0.50188	0.0690	0.0153
rs7114467	11	15414043	*INSC*	A	G	0.45957	-0.0691	0.0152

Genotyping result of 15,495 Japanese subjects were anlayzed in this study. A1 frequency of
from release 24 Hapmap JPT. *Effect size and SE of allele1 on age at menarche (year per allel
were obtained by meta-analysis after separate analyses divided by disease status.
